# Phytochemistry and Biological Activities of *Hedeoma piperita* Benth. (*Quiensabe*)

**DOI:** 10.3390/ijms26041640

**Published:** 2025-02-14

**Authors:** Jeanette Guadalupe Cárdenas-Valdovinos, Hortencia Gabriela Mena-Violante, Flor de Fátima Rosas-Cárdenas, María Valentina Angoa-Pérez, Silvia Luna-Suárez

**Affiliations:** 1 Instituto Politécnico Nacional, Centro de Investigación en Biotecnología Aplicada (CIBA-IPN), Tepetitla 90700, Tlaxcala, Mexico; jcardenasv@ipn.mx (J.G.C.-V.); frosasc@ipn.mx (F.d.F.R.-C.); 2 Instituto Politécnico Nacional, Centro Interdisciplinario de Investigación para el Desarrollo Integral Regional Unidad Michoacán (CIIDIR-IPN Unidad Michoacán), Jiquilpan 59510, Michoacán, Mexico; vangoa@ipn.mx

**Keywords:** Lamiaceae, medicinal plant, rosmarinic acid, antioxidant, antibacterial, antihypertensive, anti-inflammatory, antidiabetic

## Abstract

*Hedeoma piperita* Benth. (Lamiaceae) is a native medicinal plant from Mexico. It grows in pine, oak, and oyamel forests, as well as grasslands. In the Purépecha Plateau of Michoacán, it is called *quiensabe* and traditionally used to treat stomach pain, colic, cough, and low blood pressure, among other ailments. This study aimed to determine the phytochemical profile of infusions and ethanolic extracts of the stems and green and purple leaves of *H. piperita* collected in Cherán, Michoacán. Total phenols, flavonoids, anthocyanins, and terpenoids were analyzed using UV–visible spectrophotometry; specific phenolic acids and flavonoids were detected by high performance thin layer chromatography (HPTLC); and the volatile profile of stems, green and purple leaves was determined by solid phase microextraction in GC-MS. Biological activities such as antioxidant activities (via DPPH and ABTS methods), antihypertensive activities (angiotensin converting enzyme (ACE) inhibition), antibacterial activities (minimum inhibitory concentration (MIC), and minimum bactericidal concentration (MBC), anti-inflammatory activities (xanthine oxidase enzyme (XOD) inhibition) and antidiabetic activities (α-glucosidase enzyme inhibition) were evaluated in vitro. Results showed key compounds like rosmarinic acid, luteolin, menthone, menthol, and pulegone were identified using HPTLC and SPME/GC-MS, with organ-specific variations. Green and purple leaves infusions inhibited DPPH and ABTS^+^ by 90–99% (IC_50_ 3.3–3.8 and 7.4–11.5 µg/mL, respectively) and purple leaves infusion showed a 69.88% XOD enzyme inhibition (IC_50_ 47.991 µg/mL) and an 85.12% α-glucosidase enzyme inhibition (IC_50_ 72.49 µg/mL). Purple leaves ethanolic extract exhibited the lowest MIC and MBC against *Shigella flexneri* and ACE inhibition at 97.25% (IC_50_ 11.19 µg/mL). These results demonstrate the biological potential of *H. piperita* in the development of natural drugs and expand its use as an herbal remedy.

## 1. Introduction

Plants have been valued for their healing properties and health benefits since prehistoric times, being the basis of numerous traditional medicine systems around the world. In Indigenous communities, the ancestral knowledge of medicinal plants is deeply embedded in customs and practices, preserved through oral traditions, rituals, and daily use. More than 4000 medicinal plants have been registered in Mexico, making it the second most diverse country in the world for this type of flora. However, only 5% of these medicinal plants have been pharmacologically analyzed [1]. Lamiaceae is a botanical family comprising numerous medicinal species, such as *Hedeoma piperita* Benth., which is endemic to Mexico, reported in Hidalgo, Morelos, Estado de Mexico, and Puebla, where it is known as “tabaquillo” or “hierba de santo domingo” [2]; it is also present in the pine, oak, and oyamel forests as well as grasslands of the Purépecha plateau in Michoacán, known as *quiensabe*. *H. piperita* is a perennial, aromatic herbaceous plant, with ascending stems, branched from the base, with a height of up to 25 cm; it has white or purple flowers 6 to 8 mm in length [3]. It is widely used to relieve headaches, menstrual cramps, cold pains, and diarrhea, as well as to control low blood pressure and stomach pain; in addition, the aerial part is very popular as an infusion, consumed as a daily drink due to its pleasant flavor [4]. As a result of its broad spectrum of medicinal properties, the content of phenolic compounds, flavonoids, and terpenes in aqueous and ethanolic extracts of the foliage, as well as its antioxidant and anti-inflammatory activity in vivo, has been previously evaluated [3,5]. However, there is no detailed information on the differences in the phytochemical profile between stems and leaves separately, nor on the contribution of specific compounds to antioxidant activity. In addition, there are no studies that demonstrate its bioactive properties as being antihypertensive, antibacterial, or antidiabetic. Therefore, the objective of this study was to evaluate the phytochemical composition and antioxidant, antihypertensive, antibacterial, anti-inflammatory, and antidiabetic properties of infusions and ethanolic extracts from stems and leaves of *H. piperita* Benth.

## 2. Results

### 2.1. Phytochemical Content

The phytochemical content determined for *H. piperita* was higher in purple leaves than in the rest of the evaluated organs (Table 1). TPC, TFC, and TAC were significantly higher in ethanolic extracts than in infusions. It is important to note that this is the first report concerning the presence of anthocyanins in stems and leaves of *H. piperita*. TTC was significantly higher in the infusions than in the ethanolic extracts of all the evaluated organs, the purple leaves infusion showed a TTC of up to three times higher than that of ethanolic extract. The type of the extract and organ, as well as interaction between both factors, exerted a significant effect on the phytochemical content of *H. piperita*.

#### Phenolic Compound Detection by HPTLC

**Figure 1:**
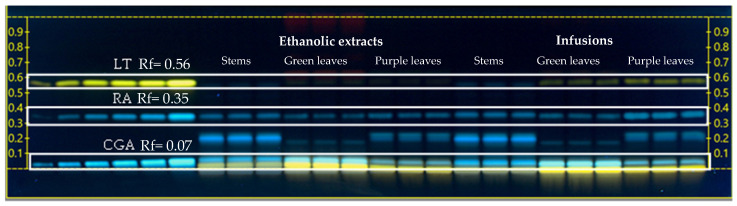
HPTLC chromatogram performed for the detection and quantification of chlorogenic acid (CGA, Rf = 0.07), rosmarinic acid (RA, Rf = 0.35), and luteolin (LT, Rf = 0.56) from stems and leaves (green and purple) for *H. piperita* infusions and ethanolic extracts, taken under UV light (366 nm) after derivatization with NP reagent. Rf, retention factor.

The presence of CGA (Rf = 0.07 ± 0.05), RA (Rf = 0.35 ± 0.02), and LT (Rf = 0.56 ± 0.012) were detected (Figure 1) in different concentrations in both types of extracts of stems and green and purple leaves of *H. piperita* (Table 2).

The CGA content determined by HPTLC was higher in infusions than in the ethanolic extracts of green leaves (12.91%), while the CGA content was similar for stems and purple leaves in both extracts of *H. piperita*. The RA content was significantly higher in infusions than in the ethanolic extracts of stems (32.98%), green leaves (49.01%), and purple leaves (64.01%). Stems presented up to three times less RA than the rest of the evaluated organs in both types of extracts. Conversely, LT in green and purple leaves was higher in infusions than in ethanolic extracts (1.7 and 1.4 times, respectively), although it was not detected in stem extracts.

### 2.2. Identification of Volatile Compounds by SPME/GC-MS

The volatile compounds identified by SPME/GC-MS in *H. piperita* are shown in Table 3. The greatest terpene diversity was found in purple leaves (23 compounds), followed by stems and green leaves (19 compounds each one). Pulegone, menthol, and menthone were the main terpenes found in the three analyzed organs. Pulegone (Rt = 10.891 ± 0.049 min) was the terpene with the highest proportion found in green leaves (39.08%), followed by purple leaves (35.89%) and stems (18.58%). Menthone (Rt = 9.989 ± 0.088 min) was found in purple leaves (36.67%), green leaves (30.4%), and stems (24.97%); while menthol (10.39 ± 0.03 min) was found in a higher proportion in stems (23%) than in green and purple leaves (~6%). γ-Terpineol was detected in the three analyzed organs from *H. piperita* (10.299 ± 0.09 min), and stems had a higher proportion (11%) compared to purple leaves (~6%). Terpenes such as isopulegol, D-sylvestrene, terpinolene, and A-bourbonene were also detected in all organs in minor proportions (less than 1%). Isodyhydrocarvone, D-limonene, copaene, and germacrene-D were found in both green and purple leaves; A-pinene, camphene, and sabinene were found in stems and green leaves.

The application of SPME/GC-MS enabled the detection of organo-specific terpenes whose presence had not been reported in *H. piperita*: verbenol, A-campholenal, endoborneol, (+)-menthol, verbenone and piperitone were found in stems; A-phellandrene and isomenthol were detected in green leaves; and finally, A-myrcene, p-menthadienol, fenchol, 5-caranol, isopulegone, piperitonone, B-copaene, cadinene, camphor, and cadinol were detected only in the purple leaves of *H. piperita*. The detection of these specific compounds in each organ evaluated may be related to the medicinal properties attributed to it and the biological activities it possesses. 

Regarding the content of terpenes in *H. piperita* (Table 4), menthone content was 36.7% higher in purple leaves compared to that found in green leaves and 75% higher than in stems. The stems contained up to 25 and 28% more menthol than green and purple leaves, respectively, while pulegone was the most abundant terpene in both types of leaves evaluated, being up to 79% higher than in stems.

### 2.3. Biological Activities

#### 2.3.1. Antioxidant Activity

The maximum antioxidant capacity was obtained in the infusions of the green and purple leaves of *H. piperita*, measured using DPPH· (89–90% inhibition, IC_50_ 3.3–3.8 µg/mL) and the ABTS method (99% inhibition, IC_50_ 7.4–11.5 µg/mL) (Table 5).

#### 2.3.2. Antioxidant Activity Through HPTLC-DPPH· and HPTLC-ABTS^+^

**Figure 2:**
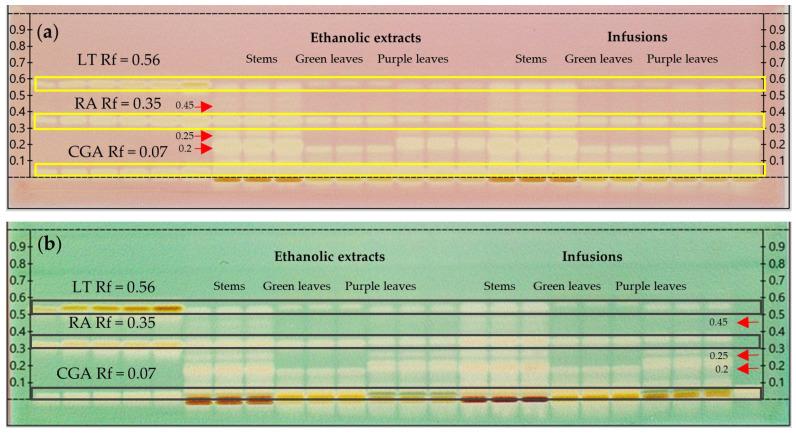
HPTLC chromatogram performed for the detection of antioxidant compounds on stems and leaves (green and purple) from *H. piperita* infusions and ethanolic extracts, taken under white light after derivatization with DPPH· (**a**) and ABTS+ (**b**) reagents.

HPTLC-DPPH· and HPTLC-ABTS^+^ techniques allow the visualization of bands in chromatogram with antioxidant potential, causing discoloration from purple to yellow (DPPH·) and from blue to yellow-white (ABTS^+^) (Figure 2a,b, respectively). All *H. piperita* extracts showed antioxidant bands at Rf = 0.07, 0.35, and 0.56, which is consistent with bands detected for CGA, RA, and LT, respectively. Additionally, unidentified antioxidant bands were observed for all extracts evaluated at Rf = 0.2; moreover, antioxidant activity was detected in infusions and the ethanolic extracts of stems and purple leaves at Rf = 0.25, and an active band of Rf = 0.45 in stems was detected in both types of extracts.

#### 2.3.3. Antihypertensive Activity

ACE maximum inhibition was shown by *H. piperita* leaves, with a greater effect in ethanolic extracts (90–97%) than in infusions (83–87%) (Table 6). The lower IC_50_ was shown by green leaves in both types of extracts. The ACE inhibition of ethanolic extracts of green and purple leaves equaled the inhibitory effect of captopril; however, the IC_50_ of this drug was in the order of hundredths, significantly higher than that in ethanolic extracts and infusions whose IC_50_ were in the order of single and double digits (4.25–51.33 µg/mL).

#### 2.3.4. Antibacterial Activity

All evaluated tissues of *H. piperita* showed antibacterial against the activities of enteropathogenic bacteria *S. enterica*, *S. flexneri*, and *E. coli* both in infusions and ethanolic extracts. The greatest antimicrobial power was found in the ethanolic extract of purple leaves, against *S. flexneri*, which was the most sensitive strain (Table 7). There were no differences between the MIC and MBC values shown by the extracts, so they should be considered inhibitors.

#### 2.3.5. Anti-Inflammatory Activity

Highest XOD inhibition was found in the purple leaf infusion of *H. piperita* (69.88%, IC_50_ 47.991 µg/mL) (Table 8), which showed similar effects as the positive control. Green leaf infusions, as well as purple and green leaf ethanolic extractions, showed an inhibition of greater than 50%, while the stems presented lower inhibition percentages, both in infusions (37.62%) and in ethanolic extracts (21.78%); this is the first report of in vitro anti-inflammatory activity of *H. piperita*, and the results show that both types of extracts evaluated are potential XOD inhibitors.

#### 2.3.6. Anti-Diabetic Activity

All *H. piperita* organs showed the inhibition of α-glucosidase enzyme activity (Table 9). Maximum inhibitory activity was found in purple leaves infusion, which had the same effect as the positive control; ethanolic extract of purple leaves, as well as infusions of stems and green leaves, showed an inhibition of 70–79%, while stems and green leaves in ethanolic extract had an inhibition of less than 65%. Purple and green leaf infusions and ethanolic extracts had an IC_50_ of less than 80 μg/mL, while the rest of extracts exhibited IC_50_ values of greater than 90 µg/mL. 

#### 2.3.7. Pearson’s Correlation

**Figure 3:**
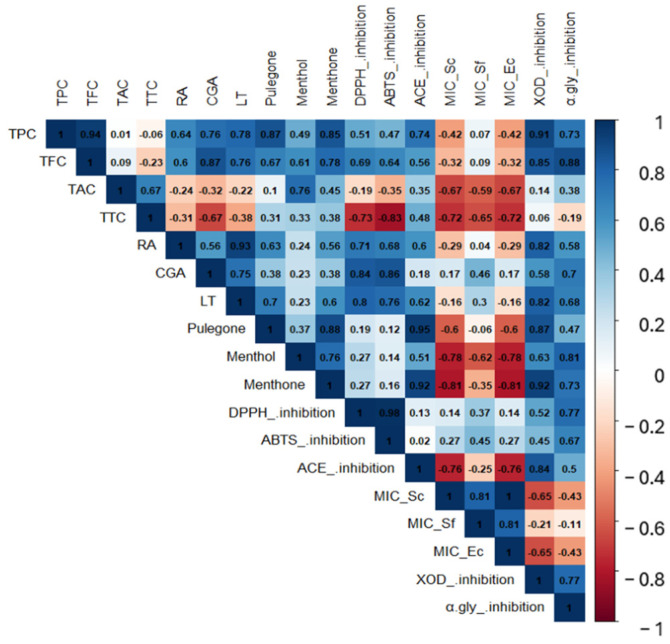
Heat map matrix of Pearson’s correlation coefficients for the presence of phytochemical compounds versus the biological activities of infusions and ethanolic extracts of *H. piperita*.

Pearson’s correlation analysis showed that the biological activities determined for ethanolic infusions and extracts of *H. piperita* are correlated with their phytochemical composition (Figure 3). TPC showed a positive correlation with XOD inhibition (0.91) and TFC showed a positive correlation with α-glucosidase inhibition (0.88). Among the specific phenolic compounds, CGA was mostly related to DPPH· and ABTS+ free radical inhibition (0.84 and 0.86, respectively). 

The majority of terpenes also showed a high correlation with biological activities. Menthone was correlated with ACE (0.92) and XOD (0.92) inhibitions, showing an inverse correlation with antibacterial effect (−0.81). Pulegone had the highest correlation with ACE inhibition (0.95) and XOD (0.87). Menthol showed a positive correlation with XOD inhibition (0.87) and α-glucosidase (0.81).

## 3. Discussion

It is important to point that literature searches were conducted in the Google Scholar, SciELO, ScienceDirect, Pubmed, Wiley Online Libraries, and Springer Link databases, as well as in university repositories in Mexico (IPN, UNAM, and the UMSNH Digital Library) in the 2021–2024 period. The keywords used were “Hedeoma piperita”, “quiensabe”, “phytochemistry”, “volatile compounds”, “traditional use”, and “biological activity”. Only two articles were found, one describing the morphotype and genetic divergence of *H. piperita* [6], as well as its cultural importance, and one on its management and conservation in an indigenous community of Michoacán [4]. In addition, four theses were found, two of which describe the phenology and ecological aspects of the plant in regard to its reproduction and use [3,7]; one describes the antidepressant activity of hydroalcoholic extracts of *H. piperita* leaves evaluated in mice [8]; and only one describes, in a general way, the effect of drying and storage time of leaves on the antioxidant and anti-inflammatory activities and the phenolic compounds and terpenes contents in *H. piperita* infusions [5]. The above supports the relevance of carrying out this research.

TPC (42.5 GAE/g DW) and TFC (23.2 QE/g DW) have been reported in infusions of the aerial parts of dehydrated *H. piperita*, at values that were, respectively, 1.7 and 2.7 times lower than those obtained in this work. TTC (18.75 mg PE/g PS) was 1.4 times higher than that found in infusions [5]. The differences observed could be due to the processing or conditioning of the sample. Raya-Ramírez [5] performed room temperature drying treatments and dark storage for 30, 60, and 90 days, observing significantly lower values of TPC and TFC during storage time of *H. piperita*. The negative impact of plant dehydration through natural convection on its nutraceutical characteristics (e.g., antioxidant activities) has been reported; therefore, freeze-drying is preferred as the drying process for medicinal plants. 

The content of phytochemicals extracted can vary between different species of the genus Hedeoma. TPC in polyphenolic extracts of oregano (*H. patens*) has been reported (99.58 ± 0.42 GAE/g) [9] at levels around 0.7 times higher than those found in *H. piperita* in this work; the authors attributed the elevated TPC of *H. patens* to the growth conditions, collection, and storage of samples. Likewise, a five-fold lower TPC has been reported in methanolic extract of aerial parts of *H. drummondii* or “pennyroyal tea” (32.36 ± 0.86 mg GAE/g) compared to that found in the purple leaf extracts of *H. piperita* in this work [10].

Phenolics compounds, flavonoids, and anthocyanins function as response molecules during stress by activating specific enzymes [11]. For example, an increase in anthocyanins production in the aerial part of *Arabidopsis thaliana* has been connected to the activation of the dihydroflavonol 4-reductase enzyme in plants subjected to stress due to N scarcity [12].

The presence of anthocyanins in stems and leaves has been reported in several species of the Lamiaceae family. Compared to the methanolic extracts of *Artemisia annua*, the TAC in ethanolic extracts of *H. piperita* was 1.7 times higher in stems (0.26 mg C3GE/g) and 2.7 times lower in purple leaves (3.59 mg C3GE/g), respectively [13]. In European lemon balm (*Melissa officinalis*), it has been hypothesized that reactive oxygen species (ROS) induced the biosynthesis of anthocyanins and terpenes to mitigate the production of reducing agents derived from a decrease in CO_2_ absorption through the Calvin cycle [14]. It has also been shown that anthocyanins accumulate in stems, leaves, and other organs, which can play direct and indirect roles as modulators of signaling cascades caused by ROS and are involved in plant growth, development, and stress responses [15].

The presence of hydroxycinnamic acids such as rosmarinic, chlorogenic, and caffeic acids have been reported in plants of the Lamiaceae family and has been related to their potential biological activity [16]. Rosmarinic acid has been reported as the most abundant phenolic acid in 20 species of Lamiaceae [17], such as in in *Salvia officinalis* (up to 230 μg/mL) and *Mentha pulegium* (up to 400 μg/mL), followed by chlorogenic and caffeic acids, hyperoside, rutin, and gallic acid. For the genus *Hedeoma*, predominant presence of RA and CGA has been reported [18], and both are the main phenolic acids identified in the methanolic extracts of *H. drummondi* [18], which is consistent with what was found in the infusions and ethanolic extracts of *H. piperita*. RA has been found in the ethanolic extract of the aerial parts of *H. mandoniana* (0.02 mg/g extract), which is eight times lower than that found in *H. piperita* [18]. In addition, RA has been found in infusions of *M. spicata*, *M. longifolia*, *M. piperita*, and *M. suaveolens* (32.34 to 43.9 mg/g) [19], where its content was 0.7 times higher than that found in infusions of purple leaves of *H. piperita*.

CGA eliminates free radicals, and it is often detected in its dimeric form (RA) [18]. RA has been detected in high concentrations in Lamiaceae plants, such as *Satureja montana*, *Origanum marjoram*, and *Thymus vulgaris*, whose aqueous extracts showed concentrations of 1.57, 0.82, and 0.471 mg/mL, respectively [20], which were up to three times lower than those obtained in the infusions of the green and purple leaves of *H. piperita*, which indicated that the traditionally consumed infusion is an excellent source of RA and CGA.

LT is a recurrent flavonoid in Lamiaceae plants such as *Thymus vulgaris*, *Origanum marjoram*, and *Salvia microphylla*, whose biological antioxidant, anti-inflammatory, anticancer, and antidiabetic activities have been evidenced [19,21].

Even though there is limited information on the presence of LT or its derivatives in the genus *Hedeoma*, the presence of LT glycosides (LT-7-O-glucuronide-3′-O-glucoside and LT-7-O-glucoronide) in chloroformic extract of Mexican oregano (*H. patens*) has been observed [22]. Similarly, LT-glucoside-glucoronide (339.58 μg/mg dry extract) has been reported in the methanolic extract of *H. patens* [23].

The SPME/GC-MS is a highly sensitive technique that allows the separation of molecules, their subsequent ionization and an accurate identification by the molecular structure elucidation [24]. For this reason, it was chosen to generate knowledge about the phytochemical profile of *H. piperita*. Additionally, this technique allows us to obtain quantitative data by providing the relative proportion of the area under the curve of each detected compound in the samples, based on the established concentrations of known standards [25,26]. 

Menthone content has been reported in *Mentha spicata* [27] (2.18 mg/g of fresh weight) at up to 42.7 times lower than that reported in the purple leaves of *H. piperita*. Menthol has been reported in the stems (4.23 mg/g) and leaves (39.06 mg/mL) of *Mentha aruensis* [28], at 5.47 and 2.37 times lower, respectively, than that found in purple stems and leaves of *H. piperita*. In addition, it was observed that pulegone content in the green and purple leaves of *H. piperita* was up to 2.7 times higher than that reported in the leaves of *Satureja macrostema* (34.05 mg/g of fresh weight) [29].

Terpenes such as pulegone, menthol, and menthone have been reported in infusions of fresh *H. piperita* leaves [5], which agreed with the results of this work. In the genus *Hedeoma*, pulegone has been reported as the main monoterpene in the essential oil of *H. pulegioides* (860 mg/mL) [30] and *H. multiflorum* (39%) [31], whose content was equal to that detected in the green leaves of *H. piperita*. In addition, the presence of menthone (23%) and piperitenone (0.1%) was detected in *H. multiflorum*, the contents of which were 1.6 and 0.4 times lower than those found in purple leaves of *H. piperita*.

The volatile profile of *H. piperita* was consistent with that reported by Muñoz et al. [18] in the essential oil of *H. mandoniana*, a Bolivian species used as an infusion to treat digestive problems and respiratory diseases. The presence of pinene, sabinene, phellandrene, myrcene, terpinene, limonene, pulegone and its derivatives, menthone and its derivatives, germacrene D, and piperitone, in different proportions, was detected, with a 68.4% similarity to the levels observed in *H. piperita*.

Pulegone (C_10_H_16_O, molecular weight 152.23 g/mol) is an oxygenated monoterpene with a strong and refreshing odor that has been reported as having antioxidant, antimicrobial, carminative, and insect repellent properties in most species of *Mentha*, specifically *M. pulegium* from which it was first isolated [32]. Pulegone is derived from terpinolene conjugation through piperitenone and is a menthone [33]. Isopulegol is derived from pulegone by reducing a ketone group to hydroxyl, resulting in a milder menthol-smelling alcohol than menthol [34]; both terpenes have been reported as antimicrobials, anti-inflammatories, antinociceptives, and analgesics [33,34].

Menthol is a monocyclic monoterpene alcohol (C_10_H_20_O, molecular weight 156.27 g/mol). It has a strong and refreshing smell and is widely used in the food industry as a flavoring agent and in the pharmaceutical industry due to its biological activity as an antimicrobial, anti-inflammatory, analgesic, antitussive, and antiviral [35]. It has eight different stereoisomers, namely (−)-menthol, (−)-neomenthol, (−)-isomenthol, and (−)-neoisomentol, and their four respective enantiomers [36]; (−)-menthol and (−)-neomenthol have been reported as components of the essential oil of *Mentha* (*M. suaveolens*, *M. pulegium*, *M. piperita* and *M. longifolium*) in proportions of 16–46% menthol and 2–9% neomenthol [37,38], which was consistent with the findings in *H. piperita* stems and green leaves.

Menthone (C_10_H_18_O, molecular weight 154.25 g/mol) is a ketone analog of menthol with a less intense minty smell [36]. It has been identified as the main constituent of essential oil of *M. suaveolens* leaves (39.4%) and has also shown immunomodulatory, antiparasitic, antibacterial, and anti-inflammatory activities [38,39,40]. 

Pinene is a bicyclic monoterpene present in plants, with the α and β enantiomers known for having antifungal, antibacterial, and anticancer activities [41]. Both have been reported in the essential oil of *M. longifolia* and *M. pulegium* (1.7–9%) in amounts up to three times higher than those found in *H. piperita* tissues [42]. Camphene, campholenal, and camphor are derivatives of the mevalonic acid pathway and exhibit antimicrobial, antitussive, antidiabetic, antiparasitic, and anticancer activities [43]. These have been reported in the essential oils of Lamiaceae family plants, for example, *M. spicata* (>3%, in leaves) [42], *Dracocephalum kotschyi* (0.3–6%) [44], and *Lavandula pedunculata* (1–7%, in trichomes) [45]; in all cases, its content was higher than that found in the different organs of *H. piperita* (<1%).

α-phellandrene, myrcene, limonene, terpinolene, p-menthadienol, and terpineol, as well as their derivatives, are olefinic P-menthanes found in stems, young leaves, and flowers, and act in the chemical defense of plants against pathogens and insects. These compounds have exhibited biological activities such as anti-inflammatory, antioxidant, soothing, and anticancer activities [46,47,48]. They also have been reported in the essential oils of *M. piperita* (<3%), *Calamintha nepeta* (<1%), and *R. officinalis* (<1%) [33,49,50].

Sylvestrene and sabinene are simple, fresh-smelling, and spicy bicyclic monoterpenes with antioxidant, anti-inflammatory, and antimicrobial activities, reported in *Salvia stenophylla* (Lamiaceae) essential oil in small proportions (0.1–0.2%), that matched those found in *H. piperita* [51]. Verbenol and verbenone are bicyclic monoterpenes distinguished by their functional groups (alcohol and ketone, respectively), whose precursor is α-pinene [52]. These have been reported among the essential oil constituents of *Rosmarinus officinalis* leaves and *Otostegia persica* (less than 1%) [50,53], which have powerful acaricides, antimicrobials, antioxidants, and anti-ischemic activities [52].

Endoborneol, fenchol, and 5-caranol are monoterpene alcohols derived from geranyl pyrophosphate that have potential as antimicrobials and antioxidants; their essential oil contents in the leaves of *Ocimum forskolei* and *Teucrium yemense* (Lamiaceae) [54] and the ethanolic extract of *R. officinalis* leaves [55] vary between 1 and 31%, up to 20 times greater than that found in the stems and purple leaves of *H. piperita*.

Dihydrocarvone is a monoterpene ketone obtained from the reduction in carvone and is present in *M. longifolia* and *M. spicata* in proportions less than 2% [42]. Piperitone and its isomer piperitenone are also common monoterpene ketones in the essential oils of *M. cervina* and *M. piperita*, although piperitone is only present in smaller amounts (0.6–6.4%) than piperitenone (3–30%) [37]; both have been reported to have antimicrobial and antifungal activities against pathogenic plant microorganisms [56].

Copaene, bourbonene, germacrene D, cadinene, and cadinol are sesquiterpenes that originate from the mevalonate and methylerythritol phosphate pathways [57]; copaene has been reported in low amounts (<1%) in the essential oils of *M. suaveolens* and *M. spicata* [56], bourbonene has been found in *C. nepeta* (>3%) [33], and germacrene D and cadinene have been identified in *M. cardiaca* and *M. spicata* (1–4.46%) [42], while cadinol, along with the other sesquiterpenes mentioned above, has been detected in the essential oil of *Marrubium vulgare* and *Thymus algeriensis* [58]. 

The observed differences in the phytochemical compositions of stems, green leaves, and purple leaves of *H. piperita* can be explained by the theory of optimal defense (ODT), which states that the distribution of chemical defense in different organs of the plant depends on the importance of these in basic functions such as growth. ODT predicts that young leaves are more important for the plant, so the greatest accumulation of phytochemical compounds involved in chemical defense must be found in them, while in more mature tissues, the accumulation of metabolites decreases due to the decrease in photosynthetic rate and nitrogen concentrations [59,60]. This behavior was reflected in the significant differences found in TPC, TFC, TAC, TTC, CGA, RA, LT, and the majority terpenes menthone, menthol, and pulegone between the stems, green leaves, and purple leaves of *H. piperita*.

The antioxidant activity of infusions of the aerial section of *H. piperita* was reported by Raya-Ramírez [5], measured by DPPH· (40%) and ABTS+ inhibition (80%), which was two times lower than that found in this research. The antioxidant activity for other Lamiaceae species such as *Stachys sylvatica* and *Stachys officinalis* has been reported, which was higher than that obtained here in infusions evaluated with DPPH· and ABTS (1.7 and 2.4 times, respectively) [61]. The results agreed with those reported by Franco-Aguirre et al. [62] in the ethanolic extracts of *M. piperita* leaves, in which higher inhibition percentages of DPPH· (70.6%) and ABTS+ (97.6%) were obtained. The differences between the two techniques could be explained by the different interactions (e.g., ionic bonds, hydrophobic interactions, and van der Waals forces) between DPPH· or ABTS+ with the components of plant extracts [62].

RA presence in infusions and acidified methanolic extracts of Lamiaceae such as mint (*M. piperita*), lemon balm (*Melissa officinalis*), and lavender (*Lavandula angustifolia*) has been reported and connected to the antioxidant capacity determined by ABTS+ cation inhibition [63]. In this sense, Truong et al. [64] stated that the antioxidant potential shown by RA in its aqueous phase involved the formal hydrogen transfer (FHT) pathway and the single electron transfer (SET) pathway, which broadens its spectrum to eliminate radicals such as superoxide and hydroperoxides, as is the case for other phenolic compounds such as anthocyanins [65]. This confirmed the results obtained in this research, since the infusions of different tissues of *H. piperita* showed greater antioxidant activity than those observed in ethanolic extracts.

The presence of terpenes has also been correlated with the antioxidant activity presented by *H. piperita extracts*. Sarikurkcu et al. [66], analyzed the antioxidant capacity of *M. pulegium* essential oil, the main components of which were pulegone (71.47%) and menthone (7.67%), and reported a lower antioxidant activity (5.96% inhibition of DPPH) in the essential oil (1 mg/mL) compared to the 94.77% inhibition shown by the methanolic mint extract, which was rich in phenolic compounds and flavonoids. Pulegone and menthone, present in other Lamiaceae such as *Satureja macrostema*, are oxygenated monoterpenes with a ketone group that give them low reactivity, which explains the low correlation between their content and antioxidant activity [67].

ACE is a fundamental enzyme of the renin–angiotensin–aldosterone system (RAAS), which is involved in the regulation of blood pressure and fluid balance in the body. ACE is responsible for converting angiotensin I into angiotensin II, a vasoconstrictor that increases blood pressure. In addition, it stimulates the secretion of aldosterone in adrenal glands, increasing the reabsorption of sodium and water in kidneys, thus raising blood pressure. ACE inhibitors substances work by blocking the conversion of angiotensin I to angiotensin II and lowering blood pressure [68]. 

ACE inhibitory activity shown by *H. piperita* agreed with that reported for different Lamiaceae plants. Cam et al. [69] showed that ACE activity was inhibited in 94% by methanolic extracts of *M. piperita*, a percentage that was found in the inhibition range shown by the ethanolic extracts of green and purple leaves of *H. piperita*. However, the IC_50_ was seven times lower in *M. piperita* (600 μg/mL) than in the ethanolic extract of green leaves of *H. piperita* (4.25 mg/mL, the most prominent IC_50_). In addition, ACE has been effectively inhibited by the hydroalcoholic extracts of *Lavandula pedunculata*, with an IC_50_ of 0.98 to 1.17 mg extract/mL [70]. The authors mentioned that enzyme inhibition was influenced by the high concentrations of RA in the extracts (up to 58 mg RA/g DW), which resulted in blood systolic pressure reduction, emulating commercial inhibitors such as captopril. The authors concluded that other compounds such as chlorogenic and caffeic acids, luteolin, and apigenin, could act synergistically with RA.

CGA and caffeic acid have been reported as potential ACE inhibitory agents and other key enzymes linked to hypertension, with an inhibition percentage of less than 50% for both phenolic acids, compared to 80% for the positive control (captopril) [71]. This inhibitory activity was attributed to interaction between the phenolic hydroxyl groups and the peptides of enzyme’s active site through hydrogen bonds, which can restrict substrate entry and the catalysis rate. 

The relationship between the structures of various flavonoids, such as LT, apigenin, rutin, quercetin, and kaempferol, and ACE inhibitory activity has been examined [72]. A total of 23 μM IC_50_ was reported for LT, followed by quercetin, rutin, kaempferol, and apigenin (43, 64, 178, and 183 μM, respectively), attributing the effects shown to influence of different structural groups that constitute the molecular skeleton of each flavonoid, including hydroxyl groups and the groups of glycosides attached to them. LT features two hydroxyls that allow it to interact with active ACE sites, like the effect of carboxylic acid from other commercial inhibitors of the enzyme, such as lisinopril. 

Terpenes present in extracts may also contribute to ACE inhibition. The major volatile compounds detected in *H. piperita* organs, such as pulegone, menthol, and menthone have been reported as potential blood pressure reducers. The hypotensive effect in hypertensive rats, in which ACE expression was reduced by treatments with pulegone, at doses up to 40 mg/kg [73], has been reported. Menthol has been reported as a potential hypotensive agent as well; thus, Demirci et al. [74] evaluated the inhibitory effect of essential oils from three different mint species (*M. arvensis*, *M. citrate*, and *M. spicata*) as well as the direct effect of menthol on ACE activity. Inhibition percentages of 33, 22, and 73% were reported for each mint species, respectively, while menthol reached an enzyme inhibition of 99.8%, results comparable to those obtained in the present research. The hypotensive effect was attributed to the presence of menthol and other terpenes found in peppermint essential oils.

The antibacterial activity of *H. piperita* agreed with that reported against enteropathogenic bacteria in several species of the Lamiaceae family. MIC reported against *E. coli* using the methanolic extract of *M. piperita* (Mahmoudi et al. [75]), which was two times higher (MIC = 50 μg DW/mL) than that found in purple leaf ethanolic extracts of *H. piperita*, while MBC recorded for extract of *M. piperita* equaled that obtained in the same ethanolic extract of purple leaves in this study (>100 μg/mL). The antimicrobial activity of *Satureja bachtiarica essential oil* against *S. flexneri* has been reported (MIC = 6.25 μg/mL) [76], being two times lower than that obtained in the ethanolic extract of the purple leaves of *H. piperita*. The observed antibacterial potential was attributed to the presence of monoterpenes such as carvacrol, which interacts with the bacterial cell membrane, facilitating its entry into the cell. In addition, a synergistic effect with other compounds, such as α-pinene, camphene, myrcene, α-terpinene, and p-cymene, present in lower concentrations, was suggested [77]. In this work, the observed antibacterial activity was inversely correlated with the phytochemical content of *H. piperita*, including its majority monoterpene composition. In this sense, pulegone has been reported as the main monoterpene associated with the antibacterial potential of the *M. pulegium* essential oil against enteropathogens such as *E. coli* (MIC 1.4 μL/mL, MBC 2.8 μL/mL) and *S. aureus* (MIC 2.8 μL/mL, MBC 5.6 μL/mL); a synergistic effect with other monoterpenes such as menthone and piperitone was also suggested [77]. These compounds were also found in the purple leaves of *H. piperita*, an organ that showed the highest antimicrobial activity against three bacteria evaluated.

Since the highest inhibitory activity of enteropathogenic bacteria evaluated was observed in the ethanolic extracts and infusions of the purple leaf of *H. piperita*, the presence of phenolic compounds, flavonoids, and anthocyanins in the extracts could be related to their antimicrobial activity. In Lamiaceae plants, the antibacterial activity of the infusions of *Teucrium arduini* leaves and flowers, against pathogens such as *Staphylococcus aureus*, *Bacillus subtilis*, *E. coli*, and *Pseudomonas aeruginosa*, has been reported. Inhibitory activity against *S. aureus* from flower infusions (MIC 16.66 mg/mL) and leaves (MIC 1.56–4.16 mg/mL), and against *B. subtilis* from leaf infusions (MIC 25–50 mg/mL), has been positively correlated with TPC and TFC [78].

In other plants, it is more common to detect the antibacterial activity associated with the presence of anthocyanins. For example, the antibacterial activity of hydroalcoholic flower extracts, as well as the anthocyanin-rich fraction of *Clitoria ternatea* (Fabaceae) against pathogens such as *S. flexneri*, *S. typhimurium*, *E. coli*, *P. aeruginosa*, *Enterococcus faecalis*, and *methicillin-resistant S. aureus* (MRSA), has been determined; showing 40 mg/mL MIC against all bacteria evaluated, with the exception of *E. coli*, which had greater sensitivity to the anthocyanin fraction (MIC 10 mg/mL) [79]. The antimicrobial activity was attributed to the presence of ternatins (acylated anthocyanins derived from delphinidin), and their synergistic action to inactivate enzymes crucial for cell division and alter the Krebs cycle was identified, which could lead to weakened cellular respiration and inadequate energy supply, with eventual bacterial death.

XOD is a crucial enzyme for nucleic acid metabolism within the body and is found in organs such as the lungs, heart, and liver; it participates in the catalyzation of purines, degrading them by oxidation into hypoxanthines and xanthines, which are then converted into uric acid, superoxide anions (O^2−^), and hydrogen peroxide (H_2_O_2_). When XOD production is inadequate, or there is a high consumption of foods rich in purines (e.g., red meat, fish, and seafood), uric acid accumulates, which can cause pathophysiological processes such as inflammatory disease, hyperuricemia, ischemic damage, and gout. In addition, elevated uric acid levels can lead to chronic cardiovascular diseases, such as hypertension or acute ischemic stroke in severe cases [80].

The anti-inflammatory activity determined by XOD inhibition has already been reported in different species of the Lamiaceae family. Hudaib et al. [81] analyzed the XOD inhibitory capacity of methanolic extracts of medicinal plants from Jordan, including some species of Lamiaceae family such as *Salvia spinosa*, *Rosmarinus officinalis*, *M. spicata*, and *Lavandula angustifolia*, at concentrations of 200 μg/mL. The percentage of XOD inhibition (22.5 to 71.5%) was similar to that found in *H. piperita* organs. Nguyen et al. [82] analyzed the anti-inflammatory activity of methanolic extracts of aerial parts of *Artemisia vulgaris* and *A. apiacea* and reported 89.3% and 57.4% XOD inhibition at a concentration of 100 μg/mL. The inhibitory activity was attributed to the presence of major flavonoids such as LT, apigenin, kaempferol, and eriodictyol, in addition to the presence of monoterpenes such as E-piperitol and β-pinene.

XOD inhibition by specific phenolic compounds and flavonoids has already been reported. Hydroxycinnamic acids such as rosmarinic, chlorogenic, and coumaric acid, as well as the flavonoids luteolin, apigenin, kaempferol, and quercetin have been reported as effective XOD inhibitors, with IC_50_ in the range of 1.5–2.38 μM [83]; thus, infusions and ethanolic extracts of *H. piperita* could be considered potential XOD inhibitors.

Bioactive compounds inhibiting XOD have been isolated and identified in infusions of *Perilla frutescens* (Lamiaceae) leaves, resulting in RA, caffeic acid, methyl rosmarinate, vinyl caffeate, and apigenin as the main XOD inhibitor compounds, with an IC_50_ in a range of 0.44 to 121.22 μM, pointing to a competitive inhibition [84]. The inhibitory effect of phenolic compounds on XOD can be explained by their interaction as proton and/or electron donors; this, in addition to involving a potential antioxidant effect, comprises processes such as the vinylation or methylation of the original phenolic compounds, which seems to promote XOD inhibitory activity [84].

α-glycosidase is a key enzyme in the digestion of complex carbohydrates, which acts by breaking them down into glucose molecules that are then absorbed in the intestine and passed into the bloodstream, thus raising blood glucose levels, which regulates the rate of glucose absorption. When the process is uncontrolled, it can contribute to the development of type 2 diabetes mellitus, one of the most common diseases today [85]. The inhibition of α-glucosidase is therefore an effective strategy to control glucose in patients with diabetes, as it helps to avoid sudden glucose spikes that complicate the management of the disease. Inhibitors such as acarbose act competitively on the enzyme’s active site, blocking its function by preventing interaction with substrates [86].

The effective inhibition of α-glucosidase by the ethanolic and aqueous extracts of 18 Lamiaceae, including *M. piperita* (50–92%), *Origanum vulgare* (50–78%), and *Satureja montana* (39–89%), has been reported [87]. The highest inhibitory activity shown by aqueous extracts agreed with that observed in *H. piperita* infusions, with inhibition percentages up to 0.93 times higher than those found in ethanolic extracts. Common phenolic compounds and flavonoids in Lamiaceae such as caffeic acid, chlorogenic acid, kaempferol, rosmarinic acid, and quercitrin may be linked to this enzyme inhibition by a competitive mechanism of action. 

The acetic extracts of leaves of *Perilla frutescens* (Lamiaceae), as well as its isolated fraction, which corresponds to RA, presented IC_50_ of 0.42 and 0.23 mg/mL, respectively; these values are up to 5.9 times lower than the IC_50_ found in this work. Likewise, the inhibition of α-glucosidase by phenolic compounds and flavonoids has been reported, and IC_50_ values of RA (0.8 mg/mL), LT (0.07 mg/mL), quercetin (0.45 mg/mL), and rutin (0.27 mg/mL) were determined, as well as their mechanisms of action as competitive inhibitors [88]. These IC_50_ values were 8.8 times higher than those shown by ethanolic extracts and infusions of *H. piperita*, which suggests that compounds diversity may promote a synergistic effect in terms of enzyme inhibition.

Terpenes present in aromatic Lamiaceae plants can exert inhibitory activity on α-glucosidase; for example, the essential oil of *M. spicata* leaves, whose described aromatic profile includes menthol, carvone, pulegone, menthone, pinene, caryophyllene, and p-menthone, showed a 54.93% inhibition (1.5 times lower than the percentage found in the purple leaf infusion of *H. piperita*) and an 0.68 mg/mL IC_50_ [89], which is 9.7 times greater than that of *H. piperita*. Likewise, a higher inhibitory activity has been connected to a high content of pulegone and menthol in the essential oil of *Thymus pubescens* [90], which could indicate the relationship between the majority presence of both terpenes in *H. piperita* organs and α-glucosidase inhibitory effects.

## 4. Materials and Methods

Absolute ethanol and methanol, Folin–Ciocalteu reagent, aluminum chloride, DPPH· (1,1-diphenyl 2-picrylhidrazyl), ABTS (2,2′-azino-bis (3-ethylbenzothiazoline-6-sulfonic acid) diammonium salt), Trolox ((±)-6-Hydroxy-2,5,7,8-tetramethylchromane-2-carboxylic acid), Captopril, MTT (3-(4,5-dimethylthiazol-2-yl)-2,5-diphenyltetrazolium bromide), natural products (NP reagent, 2-amino ethyl diphenyl borinate); phenolic compounds, flavonoids and terpenes standards: gallic acid (GA), quercetin (Q), rosmarinic acid (RA), chlorogenic acid (CGA), luteolin (LT), menthone, menthol, and pulegone; C8–C40 alkanes calibration standard; angiotensin-converting enzyme (ACE), hippuril histidyl leucine (HHL), quinoline, benzenesulfonyl chloride (BSC), Captopril; dimethyl sulfoxide (DMSO), xanthine oxidase (XOD), xanthine, allopurinol, α-glucosidase enzyme, p-nitrophenyl glucopyranoside (P-NPG), and acarbose were purchased from Sigma-Aldrich^®^ (St. Louis, MO, USA). HPLC-grade solvents, ethyl acetate, toluene, hydrochloric acid, and formic acid, were provided by J.T. Baker^®^ (Phillipsburg, NJ, USA). Mueller–Hinton (MH) culture media were supplied by Difco, BD^®^ (Franklin Lakes, NJ, USA). Silica gel plates HPTLC 60 F_254_ (20 cm × 10 cm, Art. 1.05642.0001) were supplied by Merck^®^ (Darmstadt, Germany).

Certified enteropathogenic strains *Shigella flexneri* (12022), *Salmonella enterica* subsp. *enterica* serovar Choleraesuis (10708) and *Escherichia coli* (12792) were obtained from American Type Culture Collection (ATCC).

*H. piperita* Benth. was collected in autumn, on the La Mojonera hill east of Cherán, Michoacán (19°43′12.000″ N, 101°50′38.400″ W, 2600 m above sea level, m.a.s.l.). M. in Sc. Ignacio García Ruiz taxonomically identified the plants (collection number 11894). One specimen was deposited in CIIDIR-IPN Michoacán Herbarium (CIMI) for long-term ex situ conservation. In the collected specimens, two types of leaf colorations were observed, namely green and purple; therefore, the phytochemical and biological activities evaluation of foliage included green and purple leaves.

### 4.1. Extract Preparation 

Stems, green and purple leaves were separated, freeze-dried, pulverized, and stored at −20 °C. A total of 100 mg of sample was extracted in 10 mL of ethanol and sonicated (60 Hz/30 min; Ultrasonic Bath PNKKODW, Rohs^®^, CIN, Sunnyvale, CA, USA) at room temperature (25 ± 2 °C); for infusions, 100 mg of sample was placed in 10 mL of distilled water at boiling point (100 ± 5 °C) for 10 min and cooled [91]. Both extracts were filtered (0.22 µm, Millipore^®^, Jaffrey, NH, USA), concentrated in rotavapor (RII, Buchi^®^, Flawil, St. Gallen, Switzerland) and freeze-dried (FreeZone 12, LabCONCO^®^, Kansas City, MO, USA), resuspended in 1 mL of methanol (100 mg/mL), and stored at −20 °C in amber vials until analysis [92].

### 4.2. Phytochemical Content Determination

The total phenolic content (TPC) of extracts was determined by the modified colorimetric method [93]. A total of 100 μL of sample was added to 500 μL of Folin–Ciocalteu reagent (1:10 dilution) and incubated at room temperature (25 °C ± 2) for 5 min, and 400 μL of Na_2_CO_3_ 0.7 M was added. The mixture was stirred in vortex and incubated for 30 min (25 °C ± 2). A total of 150 μL was placed in a 96-well microplate, the absorbance was measured at λ = 765 nm (PowerWave HT, Biotek Instruments, Winooski, VT, USA), the extraction solvent was used as a blank, and 8 concentrations of GA were utilized as the standard (0–10 mg) [calibration curve Abs_756nm_ = 0.0035 (GA) + 0.5996, R^2^ = 0.9841]. The results were expressed as milligrams of GA equivalents per gram of dry weight (mg GA eq/g DW).

Total flavonoid content (TFC) was determined by the colorimetric method, as reported by Woisky and Salatino [94] with some modifications. In 96-well microplates, 20 μL of sample was mixed with 20 μL of methanol and 100 µL of solution 5% AlCl_3_. The mixture was incubated for 20 min in darkness (25 °C ± 2), and absorbance was measured at λ = 425 nm using the extraction solvent as a blank and 8 concentrations of QE as the standard (0–10 mg) [calibration curve Abs_425nm_ = 0.134 (QE) + 0.0014, R^2^ = 0.9991]. The results were expressed as equivalent milligrams of QE per gram of dry weight (mg eq QE/g PS).

Total anthocyanin content (TAC) was determined by the differential pH method with some modifications [95]. A total of 200 μL of sample was added to 2.8 mL of potassium chloride (pH 1.0, 0.025 M); separately, 200 μL of sample was mixed with 2.8 mL of sodium acetate (pH 4.5, 0.4 M). The absorbance of the solutions was measured at 535 and 700 nm, and TAC was calculated with Equation (1):

(1) $$TAC\left(mg\;eq\;C3G/mg\;DW\right)=(A)(MW)(DF)(1000)/(\varepsilon \times pathlength)$$

where A is the absorbance of the diluted sample (A = (A_535nm_ − A_700nm_)_pH 1.0_ − (A_535nm_ − A_700nm_)_pH 4.5_); MW is the molecular weight 449.2 g/mol (cyanidin 3-glucoside, C3G); DF is the dilution factor (total dilution volume/added sample volume); ε is 29,600 (molar absorbance C3G); and the pathlength is 0.32 cm. The results were expressed in mg equivalents of C3G per g dry weight (mg eq C3G/g DW).

Total terpenoid content (TTC) was as reported by Lukowski et al. [96] with modifications. A total of 100 μL of sample was added to the 900 μL of chloroform, vigorously mixed in vortex and incubated at room temperature (25 °C ± 2) for 3 min. A total of 60 μL of sulfuric acid was added into a cold bath, after 1 h in darkness; the supernatant was removed, and the precipitate resuspended in 1 mL of methanol. Absorbance was read at 538 nm, methanol was used as a blank and 8 concentrations of pulegone as the standard (0–40 mg/mL) [calibration curve Abs_538nm_ = 0.041 (pulegone) + 0.0408, R^2^ = 0.9665]. The results were expressed in mg pulegone equivalents per gram of dry weight (mg PE/g DW).

#### 4.2.1. Detection and Quantification of Phenolic Compounds by HPTLC

The detection of phenolic compounds was performed by high-performance thin-layer chromatography (HPTLC), using method described by Shanaida et al. [97] with some modifications. Samples (12.5 mg/mL) and standards of chlorogenic acid, rosmarinic acid, and luteolin (CGA, RA, and LT, respectively; 100 μg/mL in MetOH) were placed using Automatic TLC Sampler 4 (ATS4, CAMAG^®^, Muttenz, Switzerland) onto a 20 × 10 cm plate, with a band spacing of 10 mm, distance from the bottom edge of 8 mm, and left side distance of 10 mm; 24 bands were utilized. A total of 3 μL of ethanolic extracts and infusions of stems, green, and purple leaves of *H. piperita* were examined in triplicate (100 nL/s). The plate was developed in an Automated Developing Chamber 2 (CAMAG) with a relative humidity of 47 ± 2% (humidity controller with potassium thiocyanate saturated solution), with a mixture of toluene, ethyl acetate, and formic acid (5:4:1, *v*/*v*/*v*). The migration distance was 50 mm and development time was 20 min. After development, the plate was dried with cold air for 5 min. After chromatographic separation, the plate was heated in a TLC Plate Heater III (CAMAG^®^) 100 °C for 5 min and derivatized in a TLC Immersion Device III (CAMAG^®^) at a vertical speed of 3 cm/s, with a 1% Natural Products (NP) solution to reveal the phenolic compounds and flavonoids (1 g of 2-aminoethyl diphenylborinate diluted in 200 mL of methanol); the immersion time was 3 s. After derivatization, the plate was heated (3 min, 100 °C) to remove excess solvent. The plates were evaluated using the TLC Visualizer Documentation System (CAMAG). All images were captured under white light and UV light at 366 nm. The data were processed by VisionCats (CAMAG^®^) version 2.4 software.

CGA, RA, and LT, were quantified by estimating the height of the determined peaks by means of a calibration curve (CGA = −6.781 × 10^−13^ x^2^ + 1.018 × 10^−6^ − 4.323 × 10^−2^, R^2^ = 0.9998, Rf = 0.062; RA= 6.949 × 10^−7^x − 2.205 × 10^−2^, R^2^ = 0.9979, Rf = 0.42; LT= 6.184 × 10^−7^x − 3.578 × 10^−2^, R^2^ = 0.9779, Rf = 0.66). Different volumes (0.1, 0.3, 0.5, 0.7, and 0.9 µL, equivalent to 10, 30, 50, 70, and 90 µg, respectively) of the standard solutions were placed in triplicate on the plates, in addition to the infusions and ethanolic extracts of each organ evaluated. The method was validated for instrumental accuracy, repeatability, specificity, and linearity.

#### 4.2.2. Volatile Compounds Identification by SPME/GC-MS

The presence of volatile compounds in *H. piperita* organs was evaluated through solid-phase microextraction by gas chromatography coupled to a mass spectrometer (SPME/GC-MS), in accordance with Raya-Ramírez [5] with some modifications. A total of 100 mg of sample in 20 cm^3^ amber glass vials was placed in a dry bath for 5 min at 40 °C (Drybath Stdrd, Thermo Scientific^®^, Waltham, MA, USA); then, a sample of volatile compounds was placed in SPME for 3 min (fiber 50/30 μm DVB/CAR/PDMS, Stableflex 24Ga, manual holder, Supelco^®^, Bellefonte, PA, USA) and was analyzed by GC-MS (Clarus 680/Clarus SQ 8T, Perkin-Elmer^®^, Waltham, MA, USA), with helium as the mobile phase (1 mL/min), using a split injection (50:1), at 250 °C in injector (capillary column Elite 5MS (30 m × 0.25 mm I.D. × 0.25 μm df)) under the following conditions: initial temperature of 50 °C, followed by 10 °C/min to 200 °C for 5 min. The run time was 26 min. The mass spectrometer was operated at a flow rate of 1 mL/min, with an ionization voltage at 70 eV, at an interface temperature of 250 °C and 230 °C, in full scan mode, and with a mass range of 40–500 *m*/*z*. Compound identification was carried out by comparing the calculated retention index (Ri) based on a homologous series of n-alkanes (C8–C40), mass spectrum, and retention time (Rt) and by matching with NIST/EPA/NIH spectral database (2017). The quantification of majoritarian terpenes (menthone, menthol, and pulegone) was performed by estimating the area of corresponding peaks by means of a 5-point calibration curve (0, 0.1, 0.2, 0.5, 0.75, and 1 mg/mL); results were expressed in mg equivalents per g dry weight (mg/g DW) [25]. The rest of the identified compounds were reported with the area under the curve of each peak (% area).

### 4.3. In Vitro Biological Activities

#### 4.3.1. Antioxidant Activity by DPPH^·^ and ABTS^·+^ Microdilution Method

Antioxidant activity was determined through the inhibition of DPPH· and ABTS^·+^ using the microdilution method [98], using dilution solvent as a blank. Results were expressed in millimol equivalents of Trolox per g dry weight (mM TE/g DW) (calibration curve at 10 concentrations: 0–20 mmol; DPPH· Abs_515nm_ = −0.0793x + 0.2178, R^2^ = 0.9577; ABTS^+^ Abs_734nm_ = −0.1058x + 1.3092, R^2^ = 0.9907) IC_50_ was determined from DPPH^·^ and ABTS^+^ response to 5 extract concentrations (0, 25, 50, 75, and 100 µg/mL) and inhibition percentage (100 μg/mL), calculated with Equation (2) [99].

(2) $$Inhibition\;\%=(1-\frac{Sample\;absorbance}{Blank\;absorbance})\times 100$$

#### 4.3.2. Antioxidant Activity by HPTLC-DPPH· and HPTLC-ABTS^+^

In addition, HPTLC plates subjected to same conditions as in Section 4.2.1, were derivatized with the methanolic solution of DPPH· and ABTS^+^ to detect the antioxidant activity of separated bands [100].

#### 4.3.3. Antihypertensive Activity by ACE Inhibition

ACE inhibitory activity was determined, as described by Chen et al. [101] with some modifications. A total of 10 μL of sample was added to 30 μL of HHL, stirred, and incubated for 5 min at 37 °C, and 20 μL ACE (0.1 U/mL) was added and incubated at 37 °C for 1 h. The reaction was stopped with HCl 1 M. A total of 30 μL of this mixture was added to 285 μL of quinoline, stirred in a vortex, and 75 μL BSC was added and incubated for 30 min in darkness. Finally, ethanol was added and gently stirred by inverting the tube. This reaction mixture was placed in a 96-well microplate and absorbance reader at 492 nm. Captopril (5 μg/mL) was used as an inhibitor control. Half-maximal inhibitory concentration (IC_50_) was determined from the ACE response to 5 extract concentrations (0, 25, 50, 75, and 100 µg/mL). The ACE inhibition percentage (100 µg/mL) was calculated using Equation (3):

(3) $$ACE\;inhibitory\;activity\left(\%\right)=\frac{Inhibitor\;control\;absorbance-Sample\;absorbance}{Inhibitor\;control\;absorbance-Blank\;absorbance}\times 100$$

#### 4.3.4. Antibacterial Activity by Microdilution Method

Minimum inhibitory concentration (MIC) and minimum bactericidal concentration (MBC) were determined by a microdilution method with some modifications [75]. In 96-well microplates, 100 μL of MH broth was added to 10 μL of bacterial inoculum (1.5 × 10^8^ UFC/mL). Sterility control (MH broth), growth control (broth + inoculum), and positive control (Ciprofloxacin, Sigma-Aldrich^®^, St. Louis, MO, USA; 1 mg/mL) were set. Samples and positive control were serially diluted (100, 50, 25, 12.5, and 6.25 μg/mL) and 50 μL was added to each well. Microplates were incubated with constant shaking (37 °C/150 rpm for 18 h, Scientific CVP-250^®^, Bohemia, NY, USA). A total of 15 μL of aqueous MTT solution (1 mg/mL) was added to wells as a growth indicator and was incubated for 2 h. The lowest concentration of unstained wells was considered MIC. MBC was determined by culturing 50 μL of samples of each well without color change on MH agar and incubating them at 37 °C for 24 h. The lowest concentration that did not produce growth after this subculturing was considered as the MBC.

#### 4.3.5. Anti-Inflammatory Activity by XOD Inhibition

Xanthine oxidase (XOD) inhibitory activity was determined using the Wee et al. [102] method with slight modifications. Infusions and ethanolic extracts were resuspended in DMSO; DMSO was used as negative control, and allopurinol (100 μg/mL) as positive control. A total of 50 μL of sample was added to 50 μL XOD (0.1 U/mL) and 650 μL potassium phosphate buffer (PBP, 50 mM, pH 7.5); the mixture was incubated at 25 °C for 10 min, and 500 μL of xanthine (0.15 mM) was added. Samples were incubated at 30 °C for 10 min, and 200 μL was added in a 96-well microplate and absorbance was read at 340 nm. IC_50_ was determined from the XOD response to 5 extract concentrations (0, 25, 50, 75, and 100 µg/mL). The XOD inhibition percentage (100 μg/mL) was calculated with Equation (2).

#### 4.3.6. Anti-Diabetic Activity by α-Glucosidase Inhibition

α-glucosidase inhibition was determined using the microplate spectrophotometric method with modifications [103]. A total of 170 μL phosphate buffer (5 mM, pH 6.8), followed by 10 μL of sample and 10 μL of glucosidase (0.4 U/mL), was placed on a microplate and incubated for 10 min at 37 °C. A total of 10 μL substrate (P-NPG, 0.5 mM) was added to wells and incubated at 37 °C for 30 min and the absorbance was read at 405 nm (Multiskan FC, ThermoFisher^®^, Vantaa, Helsinki, Finland). DMSO was used as a negative control and acarbose (5 mg/mL) as a positive control. IC_50_ was determined from α-glucosidase response to 5 extract concentrations (0, 25, 50, 75, and 100 µg/mL). The percentage of inhibition (100 µg/mL) was calculated with Equation (2) [104].

### 4.4. Statistical Analysis

All trials were performed in triplicate. An analysis of variance (ANOVA) was utilized, and the means were separated using a Tukey test (*p* ≤ 0.05). The half-maximal inhibitory concentration (IC_50_) was calculated with *Quest Graph™ IC50 Calculator*, AAT Bioquest software (https://www.aatbio.com/tools/ic50-calculator, accessed on 24 April 2024) [105]. Pearson’s correlation tests were performed with statistical software R^®^ for Windows, version 2023.12.1.

## 5. Conclusions

To the best of our knowledge, this is the first report to highlight differences in phytochemical profile and biological activities between the infusions and ethanolic extracts of the stems, green leaves, and purple leaves of *H. piperita*. It was observed that infusions exhibited higher total phenolic, flavonoid, and terpenoid contents, while ethanolic extracts stood out for having high levels of total anthocyanin content. This is also the first report of volatile profiles detected in different *H. piperita* organs. The presence of specific phenolic compounds, such as chlorogenic and rosmarinic acids; flavonoids, such as luteolin; and predominant terpenes, such as pulegone, menthol, and menthone, correlated with the antioxidant, antihypertensive, antibacterial, anti-inflammatory, and antidiabetic activities shown by *H. piperita* ethanolic extracts and infusions. These findings show the therapeutic potential of *H. piperita* for the treatment of conditions mainly related to antihypertensive and antidiabetic activities. However, further research is needed to evaluate their effects in vivo and in clinical studies, as well as to isolate the bioactive compounds responsible for the observed effects. 

## Figures and Tables

**Table 1:** Phytochemical content of *H. piperita* infusions and ethanolic extracts.

Extract	Plant Organ	TPC ^1^	TFC ^2^	TAC ^3^	TTC ^4^
Infusion	Stems	19.633 ± 2.617 d	8.119 ± 1.213 e	0.067 ± 0.006 cd	7.89 ± 1.221 a
	Green leaves	63.337 ± 2.886 b	46.636 ± 3.337 b	0.023 ± 0.007 d	10.736 ± 1.685 a
	Purple leaves	70.259 ± 3.256 a	62.671 ± 4.249 a	0.429 ± 0.035 b	13.236 ± 2.103 a
Ethanolic	Stems	19.987 ± 0.353 d	3.621 ± 0.466 e	0.439 ± 0.043 b	0.934 ± 0.1 c
	Green leaves	46.136 ± 1.239 c	15.314 ± 1.588 d	0.105 ± 0.014 c	2.493 ± 0.848 c
	Purple leaves	44.497 ± 3.14 c	28.973 ± 2.94 c	1.302 ± 0.104 a	4.004 ± 1.037 b
Significance	Extract	***	***	***	***
	Organ	***	***	***	***
	Extract–Organ	***	***	***	**

Mean ± standard deviation is shown. Two-way analysis of variance (ANOVA) was performed. Asterisks indicated significant differences (** *p* ≤ 0.01; *** *p* ≤ 0.001). Tukey’s test was performed (*n* = 9, *p* < 0.05), different letters indicated significant differences in columns. ^1^ Total phenolic content, mg GAE/g DW; ^2^ total flavonoid content, mg QE/g DW; ^3^ total anthocyanins content, mg C3GE/g DW; ^4^ total terpenoid content, mg PE/g DW.

**Table 2:** Phenolic compounds quantification in infusions and ethanolic extracts of *H. piperita* by HPTLC.

Extract	Plant Organ	CGA	RA	LT
Infusion	Stems	8.758 ± 0.449 c	19.872 ± 0.658 c	ND
	Green leaves	17.025 ± 0.581 a	27.887 ± 0.418 b	0.844 ± 0.045 a
	Purple leaves	6.718 ± 0.908 d	45.14 ± 0.393 a	0.648 ± 0.01 a
Ethanolic	Stems	8.064 ± 0.332 c	13.317 ± 0.447 e	ND
	Green leaves	14.827 ± 1.09 b	14.217 ± 0.55 e	0.468 ± 0.006 b
	Purple leaves	6.271 ± 0.553 d	16.252 ± 0.517 d	0.488 ± 0.018 b
Significance	Extract	***	***	***
	Organ	***	***	***
	Extract-Organ	**	***	***

Mean ± standard deviation is shown (mg/g DW). A two-way analysis of variance (ANOVA) was performed. Asterisks indicated significant differences (** *p* ≤ 0.01; *** *p* ≤ 0.001). Tukey’s test was performed (*n* = 9, *p* < 0.05); different letters indicate significant differences in columns. CGA = chlorogenic acid; RA = rosmarinic acid; LT = luteolin.

**Table 3:** Volatile compounds in *H. piperita* identified by SPME/GC-MS.

Peak Number	Rt ^1^	Ri ^2^	Compound Name	Plant Organ (Peak Area %)		
				Stems	Green Leaves	Purple Leaves
1	6.404 ± 0.022	954	A-pinene	2.772	0.294	ND
2	6.763 ± 0.01	983	Camphene	0.139	0.191	ND
3	7.238 ± 0.03	1022	A-phellandrene	ND	0.84	ND
4	7.296 ± 0.024	1027	B-pinene	ND	2.88	0.48
5	7.548 ± 0.015	1047	A-myrcene	ND	ND	0.26
6	8.202 ± 0.009	1101	D-limonene	ND	1.554	1.689
7	8.828 ± 0.005	1152	D-sylvestrene	0.236	0.223	0.321
8	9.022 ± 0.012	1167	Terpinolene	0.104	1.04	0.146
9	9.257 ± 0.013	1187	Sabinene	0.703	0.62	ND
10	9.309 ± 0.018	1191	p-menthadienol	ND	ND	0.28
11	9.356 ± 0.021	1195	Verbenol	7.3	ND	ND
12	9.569 ± 0.010	1212	A-campholenal	0.286	ND	ND
13	9.857 ± 0.026	1235	Isopulegol	0.54	0.501	0.499
**14**	**9.989 ± 0.088**	**1246**	**Menthone**	**24.967**	**30.404**	**36.662**
15	10.128 ± 0.02	1258	Endo-borneol	2.656	ND	ND
16	10.177 ± 0.013	1260	Dyhidrocarvone	ND	3.699	3.549
17	10.185 ± 0.042	1262	Neomenthol	1.139	ND	ND
18	10.222 ± 0.009	1265	Isomenthol	ND	1.824	ND
19	10.235 ± 0.003	1267	Fenchol	ND	ND	1.441
20	10.299 ± 0.09	1272	γ-Terpineol	11.19	6.749	6.841
**21**	**10.39 ± 0.03**	**1279**	**Menthol**	**23.487**	**6.839**	**6.339**
22	10.521 ± 0.005	1290	Verbenone	2.99	ND	ND
23	10.66 ± 0.043	1301	5-caranol	ND	ND	0.849
24	10.778 ± 0.001	1311	4-carene	1.091	1.541	ND
**25**	**10.891 ± 0.049**	**1320**	**Pulegone**	**18.585**	**39.088**	**35.897**
26	10.940 ± 0.009	1324	Isopulegone	ND	ND	5.41
27	11.011 ± 0.051	1330	Piperitone	1.289	ND	ND
28	11.84 ± 0.008	1397	Piperitenone	ND	ND	1.237
29	12.171 ± 0.01	1424	Copaene	ND	0.25	0.58
30	12.254 ± 0.006	1431	A-bourbonene	0.084	0.103	0.29
31	12.659 ± 0.02	1464	B-copaene	ND	ND	0.144
32	13.041 ± 0.021	1495	Cadinene	ND	ND	0.23
33	13.102 ± 0.01	1500	Germacrene-D	0.1	0.239	0.84
34	13.353 ± 0.012	1520	Camphor	ND	ND	0.23
35	13.382 ± 0.013	1523	Cadinol	ND	ND	0.56
		Total (%)		99.698	99.81	99.622

^1^ Retention time (min ± SD). ^2^ Retention index relative to C8–C40 alkanes calibration standard. ND = compound not detected. Compounds in bold were considered as majority.

**Table 4:** Major terpenes quantification in *H. piperita* identified by SPME/GC-MS.

Terpenes	Rt ^1^	Ri ^2^	Plant Organ		
			Stems	Green Leaves	Purple Leaves
Menthone ^3^	9.947	1243	23.143 ± 3.646 c	58.639 ± 5.438 b	92.674 ± 1.64 a
Menthol ^4^	10.349	1276	93.212 ± 0.381 a	70.665 ± 6.283 b	66.833 ± 5.161 b
Pulegone ^5^	10.86	1319	19.151 ± 2.965 b	94.49 ± 2.846 a	94.158 ± 4.684 a

Mean ± standard deviation is shown (mg/g DW). Tukey’s test was performed (*n* = 3, *p* < 0.05), different letters indicate significant differences in columns. ^1^ Retention time (min). ^2^ Retention index relative to C8–C40 alkanes calibration standard. Calibration curves: ^3^ menthone = 282,157[x] + 5.5754 × 10^6^, R^2^ = 0.9985; ^4^ menthol = 126,440[x] + 2.62794 × 10^8^, R^2^ = 0.9427; ^5^ pulegone= 264,135[x] + 2.77727 × 10^6^, R^2^ = 0.9987.

**Table 5:** Antioxidant activity of infusions and ethanolic extracts of *H. piperita*.

Extract	Plant Organ	DPPH· ^1^	Inhibition % ^2^	IC_50_^3^	ABTS^+ 1^	Inhibition % ^2^	IC_50_^3^
Infusion	Stems	0.220 ± 0.006 b	80.442	7.773	11.066 ± 0.018 ab	91.776	12.152
	Green leaves	0.244 ± 0.005 a	89.090	3.819	11.926 ± 0.007 a	98.847	11.581
	Purple leaves	0.248 ± 0.004 a	90.498	3.381	11.953 ± 0.011 a	99.066	7.431
Ethanolic	Stems	0.002 ± 0.009 c	2.112	77.809	0.709 ± 0.039 d	6.650	69.449
	Green leaves	0.025 ± 0.011 c	10.407	79.152	1.723 ± 0.049 c	14.983	39.746
	Purple leaves	0.115 ± 0.011 bc	42.735	73.074	3.612 ± 0.039 b	30.505	48.241

Mean ± standard deviation is shown. Analysis of variance (ANOVA) and Tukey’s test was performed (*n* = 9, *p* < 0.05), different letters indicate significant differences in columns. ^1^ mM ET/g DW; ^2^ Response observed with an extract concentration of 100 µg/mL; ^3^ µg/mL.

**Table 6:** Antihypertensive activity of infusions and ethanolic extracts of *H. piperita*.

Extract	Plant Organ	ACE Inhibition % ^1^	IC_50_^2^
Infusion	Stems	59.332 ± 0.023 c	51.33
	Green leaves	87.314 ± 0.038 b	4.25
	Purple leaves	83.331 ± 0.012 b	41.80
Ethanolic	Stems	54.893 ± 0.006 c	12.18
	Green leaves	90.986 ± 0.105 ab	9.08
	Purple leaves	97.258 ± 0.008 a	11.19
(+) Control	Captopril	100 a	0.04

Mean ± standard deviation is shown. Analysis of variance (ANOVA) and Tukey’s test was performed (*n* = 9, *p* < 0.05), different letters indicate significant differences in columns. ^1^ Response observed with an extract concentration of 100 µg/mL; ^2^ µg/mL.

**Table 7:** Antibacterial activity of infusions and ethanolic extracts of *H. piperita* against enteropathogenic bacteria.

Extract	Plant Organ	Bacteria Evaluated					
		*S. enterica*		*S. flexneri*		*E. coli*	
		MIC	MBC	MIC	MBC	MIC	MBC
Infusion	Stems	100	>100	50	>100	100	>100
	Green leaves	100	>100	100	>100	100	>100
	Purple leaves	50	100	25	100	50	100
Ethanolic	Stems	100	>100	50	>100	>100	>100
	Green leaves	50	>100	25	>100	50	100
	Purple leaves	25	100	12.5	100	25	>100
(+) Control	Ciprofloxacin	1.56	3.12	0.78	1.56	1.56	3.12

Results were expressed as µg/mL.

**Table 8:** Anti-inflammatory activity of infusions and ethanolic extracts of *H. piperita*.

Extract	Plant Organ	XOD Inhibition % ^1^	IC_50_ ^2^
Infusion	Stems	37.624 ± 0.603 c	83.209
	Green leaves	56.931 ± 1.026 b	67.803
	Purple leaves	69.884 ± 0.732 a	47.991
Ethanolic	Stems	22.031 ± 1.557 d	85.662
	Green leaves	55.363 ± 1.578 b	81.969
	Purple leaves	57.426 ± 0.539 b	74.194
(+) Control	Allopurinol	72.195 ± 3.139 a	0.756

Mean ± standard deviation is shown. Analysis of variance (ANOVA) and Tukey’s test was performed (*n* = 9, *p* < 0.05); different letters indicate significant differences in columns. ^1^ Response observed with an extract concentration of 100 µg/mL: ^2^ µg/mL.

**Table 9:** Anti-diabetic activity of infusions and ethanolic extracts of *H. piperita*.

Extract	Plant Organ	α-Glycosidase Inhibition % ^1^	IC_50_ ^2^
Infusion	Stems	70.725 ± 0.534 d	96.464
	Green leaves	76.463 ± 0.568 c	76.648
	Purple leaves	85.12 ± 1.09 a	72.49
Ethanolic	Stems	63.153 ± 0.764 e	107.132
	Green leaves	64.558 ± 1.022 e	107.78
	Purple leaves	79.156 ± 0.797 b	70.427
(+) Control	Acarbose	84.228 ± 0.742 a	3.535

Mean ± standard deviation is shown. Analysis of variance (ANOVA) and Tukey’s test was performed (*n* = 9, *p* < 0.05), different letters indicate significant differences in columns. ^1^ Response observed with an extract concentration of 100 µg/mL; ^2^ µg/mL.

## Data Availability

The original contributions presented in this study are included in the article. Further inquiries can be directed to the corresponding author(s).
